# Off-Label Sildenafil Citrate as a Rescue Therapy for Areolar Venous Congestion Post–Breast Reduction: Case Series and Outcomes

**DOI:** 10.3390/life15111644

**Published:** 2025-10-22

**Authors:** Stefana Catalina Bilha, Eloi Ramelli, Charline Huttin, Simona Mihaela Hogas, Patrick Ringenbach, Dumitru D. Branisteanu, Ileana Katerina Ioniuc, Ionut-Raducu Popescu, Mihaela Elena Nastasa, Mihai Hogas

**Affiliations:** 1 Endocrinology Department, Grigore T. Popa University of Medicine and Pharmacy, 700115 Iasi, Romania; stefana.bilha@umfiasi.ro; 2 Department of Plastic, Reconstructive and Aesthetic Surgery, Hautepierre University Hospital, 67200 Strasbourg, France; eloi.ramelli@chru-strasbourg.fr (E.R.); charline.huttin@chru-strasbourg.fr (C.H.); 3 Nephrology Department, Grigore T. Popa University of Medicine and Pharmacy, 700115 Iasi, Romania; 4 Department of Plastic, Reconstructive and Aesthetic Surgery, GHR Mulhouse, 68100 Mulhouse, France; ringenbach@ghrmsa.fr (P.R.); mihaela.nastasa@ghrmsa.fr (M.E.N.); 5 Department of Medicine, Charles E. Smith College of Medicine, Florida Atlantic University, Boca Raton, FL 33431, USA; dbranisteanu@fau.edu; 6 Department of Mother and Child, Grigore T. Popa University of Medicine and Pharmacy, 700115 Iasi, Romania; ileana.ioniuc@umfiasi.ro; 7 Physiology Department, Grigore T. Popa University of Medicine and Pharmacy, 700115 Iasi, Romania; ionut-raducu.popescu@umfiasi.ro (I.-R.P.); mihai.hogas@umfiasi.ro (M.H.)

**Keywords:** sildenafil, off-label, areolar distress

## Abstract

**Background:** The nipple–areolar complex (NAC) necrosis is a rare yet significant complication following breast reduction surgery, primarily linked to compromised vascularization of the NAC, particularly in nipple-sparing techniques. While multiple therapeutic strategies have been described in the postoperative setting, their application remains limited in clinical practice. **Case series:** We report on three patients that developed early NAC venous distress following bilateral breast reduction. Following discussion of therapeutic options—ranging from conventional methods such as scarification and local heparin therapy to off-label pharmacologic treatment—all three patients received oral sildenafil citrate (20 mg every 6 h), after excluding contraindications and obtaining informed consent. In two patients, sildenafil alone was sufficient to restore venous outflow and salvage the NAC. In one case, adjunct scarification and heparin therapy were necessary due to partial response. No arterial damage or major adverse effects related to sildenafil were observed during the postoperative period. **Conclusions**: This small series supports the potential use of off-label sildenafil citrate as a valuable, non-invasive pharmacologic option for treating postoperative areolar venous congestion. Its favorable safety profile, widespread hospital availability, and mechanism of enhancing microvascular circulation make it a promising adjunct in managing this complication. Further studies are warranted to standardize its use and determine its place within a broader postoperative management spectrum of compromised vascularization in breast surgery.

## 1. Introduction

Nipple-areolar complex (NAC) necrosis, a rare but serious complication of breast reduction surgery, occurs in approximately 0.7% to 5.9% of patients and is associated with inadequate vascularization of the NAC, particularly during nipple-sparing reduction mammoplasty techniques [1]. Various risk factors promote NAC, including active smoking, older age, large mastectomy specimen weight, comorbidities such as obesity, diabetes, and hypertension, as well as local factors like prior surgery or radiotherapy [2,3]. Peri-areola incision was also found to increase the risk for NAC ischemia/necrosis [2]. Arterial damage during breast reduction surgery is often the result of devascularization of the nipple flap, which is critical for maintaining the viability of the NAC [4]. Conversely, venous damage tends to occur when there is excessive plication of the pedicle, particularly in cases involving areolar transpositions greater than 18 cm or reduction of more than 2000 g of breast tissue [4,5]. Additionally, postoperative hematomas can create intrinsic congestion, further compromising venous drainage and increasing the risk of NAC necrosis [6].

Early detection and prompt treatment are key to preserving the areola after complications. Although various strategies exist for effectively managing postoperative areolar distress, their use may still be limited in the clinical setting [7].

Leech therapy, while established to potentially mitigate venous insufficiency and improve outcomes in microsurgery, may not always be available in urgent situations and can introduce risks of infection [7,8]. Negative pressure wound therapy (NPWT) can promote granulation tissue formation and reduce infection rates; however, its effectiveness may be limited by factors such as the extent of vascular damage in the NAC, the timing of intervention, and patient-specific factors including advanced age and underlying health conditions [5]. Scarification and delayed areolar suturing, although documented in some contexts, can be traumatic and require frequent nursing care, which may complicate patient recovery [9,10]. Furthermore, many local measures, such as the use of nitroglycerin cream or heat application, necessitate regular administration and do not guarantee consistent results across different patient scenarios [5]. Therefore, while these techniques may have their merits in specific contexts, their variable application and potential for discomfort underscore the need for individualized treatment strategies that minimize trauma and optimize recovery.

Sildenafil citrate is a selective phosphodiesterase type 5 (PDE5) inhibitor that primarily facilitates smooth muscle relaxation and vasodilation by enhancing levels of cyclic guanosine monophosphate (cGMP) [11]. Originally approved for erectile dysfunction and pulmonary arterial hypertension (PAH) [11], sildenafil has since shown promise in both experimental and short-term clinical studies in cardiovascular health [12] and wound healing [13]. Evidence from animal models and small clinical reports suggests PDE-5 inhibitors can enhance flap perfusion and reduce necrosis, supporting cautious adjunctive use [14,15,16,17,18].

In this manuscript we report three cases of areolar venous distress following breast reduction surgery early managed with off-label administration of sildenafil.

## 2. Case Report

We report three patients aged between 26 and 42 years old who underwent breast reduction surgery in the Plastic, Reconstructive, and Esthetic Surgery Department of the Emile Muller Hospital in Mulhouse (France) and developed early areolar venous insufficiency. Areolar venous distress was defined clinically as early postoperative bluish–purplish discoloration of the NAC with venous engorgement and brisk capillary refill <2 s after gentle blanching using a transparent spatula/cotton tip (two readings per side, averaged with a stopwatch). This was differentiated from presumed arterial compromise (pallor/coolness with delayed refill >3–4 s). After discussing treatment options (classic empiric interventions such as scarification of the areola-mammary plaque and local heparin therapy, or the “off-label” administration of sildenafil), and in the absence of contraindications to treatment with sildenafil (such as severe hepatic insufficiency, hypotension, history of stroke, history of myocardial infarction, or concomitant nitrate use), all three patients provided informed consent and were started on sildenafil 20 mg every 6 h. All patients also received amoxicillin–clavulanate 1 g every 12 h for 7 days, acetaminophen 500 mg every 6 h for analgesia, and standard wound care with soft-silicone foam dressings for infection prevention. General characteristics of the patients as well as their management are detailed in Table 1.

**Figure 1:**
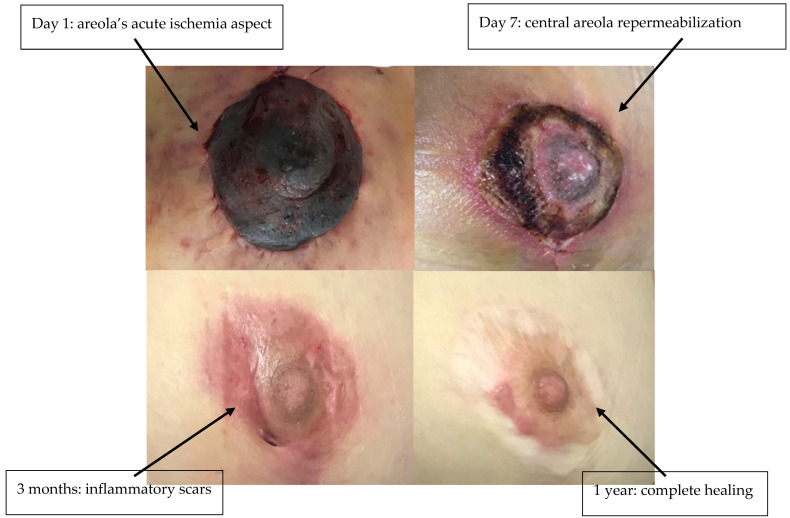
Patient 1—before and after sildenafil citrate.

**Figure 2:**
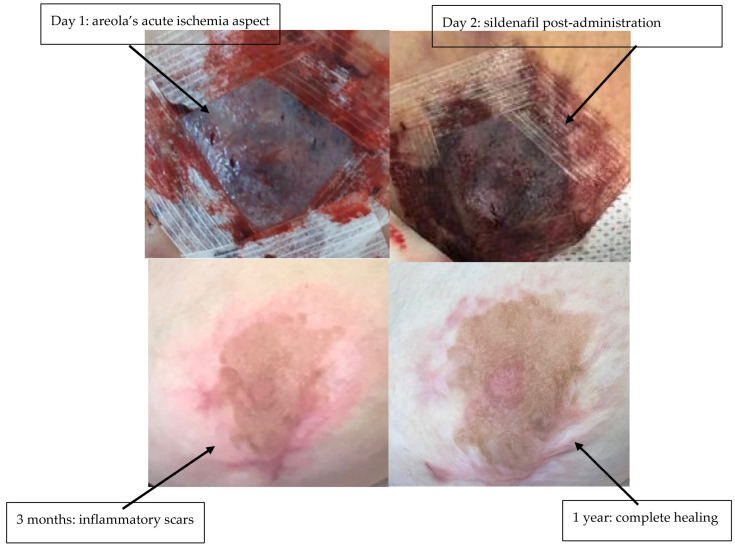
Patient 2—before and after sildenafil citrate.

**Figure 3:**
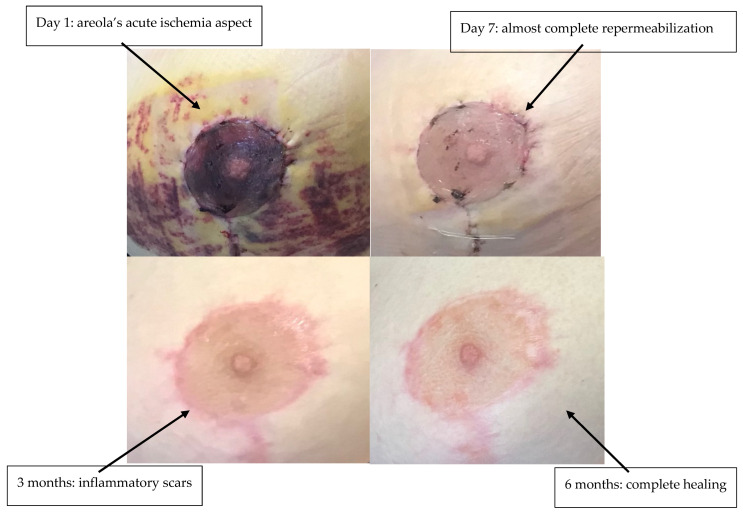
Patient 3—before and after sildenafil citrate.

Clinical evaluation was performed every 6 h. If there was no improvement after 12 h, sildenafil treatment was combined with areolar plaque scarification and local heparin therapy. Sildenafil citrate treatment was stopped after 2 consecutive evaluations observing normalization of NAC perfusion certified by normalization of the skin pulse (3 s). Total duration of treatment was 5 days in all three patients. For each NAC, viable areolar surface area at discharge was quantified by 2-D planimetry on standardized photographs (areolar border traced; necrotic/ischemic zones outlined). ‘Areolar rescue (%)’ = viable areolar area ÷ total areolar area × 100. Treatment with sildenafil citrate allowed the rescue of three NAC plaques (areolar rescue 95%, Table 1) in the process of necrosis due to suffering from venous congestion (Figure 1, Figure 2 and Figure 3). Patient 2 required additional scarification with local heparin therapy due to insufficient efficacy of sildenafil citrate (Figure 2). Patients were followed up to 1 year postoperative. There was no late necrosis in any of the patients, while patients 1 and 2 had minor periareolar post-inflammatory pigmentary sequelae at 1-year follow-up (Figure 1, Figure 2 and Figure 3). We did not observe any cases of suffering from arterial origin during the follow-up period. All patients experienced mild hot flashes and palpitations, which did not require discontinuation of treatment and resolved spontaneously after cessation.

## 3. Discussion

NAC necrosis is a dreaded complication of reduction mammoplasty [19]. To our knowledge, this is the first case series to describe off-label sildenafil citrate for managing early venous areolar distress after reduction mammoplasty, with favorable outcomes and acceptable tolerability.

Intraoperative NAC prevention requires precise pedicle planning, preservation of venous drainage, and prompt adjustments if perfusion appears compromised, while immediate postoperative recognition should trigger timely interventions such as suture loosening, delayed closure, or conversion to a free nipple graft when indicated; late necrosis is managed with supportive care [19,20]. In the absence of specific guidelines, management is at the discretion of the surgical team.

Standard rescue options include topical nitroglycerin, leech therapy, and NPWT. A meta-analysis published in 2020 by Wang et al. [21] concluded that topical nitroglycerin significantly lowers mastectomy skin-flap necrosis (including full-thickness) and debridement rates without increasing drug-related adverse events, while showing no clear effect on superficial necrosis or infection. In contrast, leech therapy directly addresses venous congestion by providing a temporary venous outflow; it is FDA-recognized for salvage of congested tissue but requires close monitoring for anemia and Aeromonas infection with appropriate antimicrobial prophylaxis [22,22,23]. NPWT can serve as an adjunct to promote perfusion and angiogenesis, thereby reducing flap necrosis [24,25,26]. A recent systematic review and meta-analysis of NPWT in breast surgery found reduced wound dehiscence and possible reductions in SSI, hematoma, seroma, and skin/wound necrosis; however, results are uncertain and varied in statistical significance [27].

Flap surgery may specifically benefit from PDE5 inhibition via improved blood flow to target tissues. Sildenafil citrate, part of the PDE5 inhibitor class, exerts its therapeutic effects predominantly through the inhibition of the enzyme phosphodiesterase type 5. This leads to increased levels of cyclic guanosine monophosphate (cGMP), which facilitates the relaxation of smooth muscle cells and vasodilation in various vascular beds, including the pulmonary and penile circulations, thereby improving blood flow and potentially reducing pulmonary vascular resistance [28]. The mechanism is initiated following the release of nitric oxide (NO), which activates soluble guanylate cyclase (sGC) to convert GTP into cGMP—resulting in a downstream cascade that involves the activation of protein kinase G (PKG). PKG mediates smooth muscle relaxation by influencing calcium dynamics and inhibiting phospholamban and L-type calcium channels, thereby lowering intracellular calcium levels and enhancing muscle relaxation [29]. Pleiotropic effects of sildenafil also include reduction in oxidative stress and DNA damage and anti-inflammatory properties via fatty acid decrease [30,31,32].

The areolar region benefits from a rich vascular supply formed by the dermal and subdermal plexuses derived from several arteries, including the lateral thoracic and internal thoracic arteries. By promoting vasodilation and increasing blood flow to the areolar muscle, a concentric smooth muscle 1 to 1.8 mm thick that represents the vascular support for the NAC and allows involuntary nipple erection and areola contraction, sildenafil could potentially improve overall vascular flow in the region [33,34].

Preclinical work consistently shows greater viable flap area and less distal necrosis with systemic PDE5 inhibition in random-pattern skin flaps, including classic rat models and studies in nicotine-exposed or diabetic settings [14,15,16,35,36]. Indeed, sildenafil increased vascular lumen count, vascular density and neutrophil influx in animal models [36,37]. Limited clinical case reports describe judicious postoperative use of PDE inhibitors to optimize flap circulation. In a retrospective case series of 11 tobacco smokers (17 local facial flaps) given postoperative sildenafil, Pfaff et al. [17] found only one distal-margin necrosis, suggesting improved flap survival versus expectations. Matin et al. [18] reported ischemic discoloration of facial interpolation flaps in two high-risk active smoker patients that received tadalafil 5 mg daily for 3 days, with visible improvement in flap color/viability by day 7 and satisfactory cosmetic outcomes on follow-up.

Due to its good safety profile and high tolerance, sildenafil citrate has garnered attention for various off-label uses, including the pediatric management of PAH [38], intrauterine growth restriction (IUGR) [39], persistent PAH after left ventricular assist device implantation [40], ventilation-perfusion mismatch in COVID-19 patients [41,42], pregnancy for reducing intrapartum fetal distress and preeclampsia [43], microcirculatory alterations in diabetic patients [44], improvement in lower urinary tract symptoms (LUTS) in men [45] or Alzheimer’s disease [46].

Sildenafil citrate is marketed as an oral tablet, oral suspension, and injection for intravenous use. Bioavailability of the molecule is rapid, with a maximum systemic concentration between 30 min and two hours after ingestion. The half-life of sildenafil is 4 h. Sildenafil citrate may cause minor adverse effects related to peripheral vasodilation (e.g., facial erythrosis, headache, dyspepsia, nasal congestion, dizziness, nausea, flushing, visual disturbance, cyanopsia and blurred vision) and is contraindicated in cases of severe hepatic insufficiency, hypotension, history of stroke, history of myocardial infarction or concomitant use of nitrates. In the context of erectile dysfunction, it is not recommended to take more than one dose per day, and the dosage varies according to the patient’s tolerance. In pulmonary arterial hypertension, no additional side effects have been described for recommendations of 3 tablets of 20 mg per day, but it is not recommended to exceed 80 mg per day over the long term [47].

Our data suggest the potentially beneficial off-label use of sildenafil citrate in managing periareolar distress occurring after reduction mammoplasty, in relation to enhancing vascular perfusion and promoting physiological responses in breast tissue. The administration of sildenafil 20 mg 4 times daily led to the rescue of three necrotic NAC plaques, with only one of them also needing scarification with local heparin therapy, without any notable side effects. A uniform postoperative regimen—soft-silicone foam dressings, acetaminophen analgesia, and a 7-day amoxicillin–clavulanate course—may have modulated perfusion, infection risk, and comfort, potentially interacting with and contributing to the observed efficacy of sildenafil. Also, we acknowledge that bedside clinical evaluation without an NAC-specific validated device represents a study limitation. Given the small, single-center retrospective series without a control group and with short follow-up, our results are hypothesis-generating only and require confirmation in adequately powered, prospective randomized trials with standardized protocols and objective perfusion endpoints.

## 4. Conclusions

In conclusion, this is the first report of sildenafil off-label use for the management of NAC venous distress following reduction mammoplasty, describing favorable outcomes. While the observed therapeutic benefit combined with the drug’s known pharmacological profile, costs, availability and safety are promising, comprehensive studies and controlled trials are necessary to validate efficacy, establish optimal dosing, and assess safety in this new setting.

## Figures and Tables

**Table 1:** Patient’s characteristics and management.

Patient	1	2	3
Age (years)	42	26	30
BMI (kg/m^2^)	29	26.74	25.8
Smoking	No	No	No
Diabetes mellitus	No	No	No
Arterial hypertension	No	No	No
Breast delta (resection weight)	Right side: 1009 g Left side: 1049 g	Right side: 855 g Left side: 819 g	Right side: 635 g Left side: 1008 g
Postoperative time before management of areolar pain	24 h	24 h	24 h
Duration of treatment	5 days	5 days	5 days
Side effects	Hot flashes, palpitations	Hot flashes, palpitations	Hot flashes, palpitations
Areolar rescue	95%	95%	95%

BMI = body mass index.

## Data Availability

The original contributions presented in the study are included in the article. Further inquiries can be directed to the corresponding author.
